# Description of *Campylobacter jejuni* Bf, an atypical aero-tolerant strain

**DOI:** 10.1186/s13099-015-0077-x

**Published:** 2015-11-19

**Authors:** Ramila Cristiane Rodrigues, Anne-Lise Pocheron, Mathieu Hernould, Nabila Haddad, Odile Tresse, Jean-Michel Cappelier

**Affiliations:** ONIRIS National College of Veterinary Medicine, Food Science and Engineering, Route de Gachet - La Chantrerie, BP 40706, 44307 Nantes Cedex 03, France; Institut National de la Recherche Agronomique (INRA), Rue de la Geraudière, 44000 Nantes, France

**Keywords:** *Campylobacter jejuni*, Oxygen tolerance, Hydrogen peroxide, Paraquat, Cell shape

## Abstract

**Background:**

*Campylobacter jejuni* is a leading cause of bacterial enteritis worldwide. This microaerophilic bacterium can survive in aerobic environments, suggesting it has protective mechanisms against oxidative stress. The clinical *C. jejuni* Bf strain is characterized by an increased resistance to oxygen. This study aimed to characterize the behavior of the clinical *C. jejuni* Bf strain under an aerobic atmosphere and in response to ROS-promoter agents.

**Methods:**

Growth was studied in both aerobic and microaerobic conditions using classic cultivable methods. Electronic microscopy and *mreB* gene expression were used to evaluate the morphology of this strain under aerobic conditions. The survival under oxidative stress was tested in the presence of different concentrations of hydrogen peroxide (H_2_O_2_) and paraquat (PQ).

**Results:**

The results showed that *C. jejuni* Bf strain can grow aerobically, unlike other strains of *C. jejuni* tested. Cells of *C. jejuni* Bf exposed to oxidative stress presented changes in morphology and the gene *mreB*, responsible for maintaining the bacillary cell morphology, was down-expressed. In aerobically acclimated conditions, *C. jejuni* Bf exhibited a higher survival rate of 52 % in the presence of H_2_O_2_ (1 mM) compared to the reference strain NCTC 11168. Concentrations above 1 mM PQ were lethal for the reference strain but not for *C. jejuni* Bf.

**Conclusions:**

Taken together, these data highlight the resistance to oxidative stress conditions of *C. jejuni* Bf, indicating that this microorganism seems more adapted to survival in hostile environmental conditions.

**Electronic supplementary material:**

The online version of this article (doi:10.1186/s13099-015-0077-x) contains supplementary material, which is available to authorized users.

## Background

*Campylobacter jejuni* was the most commonly reported bacteria associated with human intestinal infections in the European Union (EU) in 2013 [1]. In 2013, 64.8 confirmed campylobacteriosis cases per 100,000 inhabitants were reported in the EU, exceeding the number of salmonellosis cases with a notification rate of 20.4 per 100,000 population [1]. *C. jejuni* was isolated in 80.6 % of confirmed cases of campylobacteriosis [1]. In the United States, this pathogen was the second most commonly reported bacterial pathogen (behind *Salmonella*) with 6621 infections in 2013 and an incidence of 13.82 cases per 100,000 population [2]. *C. jejuni* is described as a non-spore-forming Gram-negative bacterium, highly motile, with a spiral shape [3, 4].

In most cases, people infected by *C. jejuni* often have watery diarrhea, cramp, abdominal pain and fever, which may be accompanied by nausea and vomiting. Campylobacteriosis may also result in post-infection complications such as Guillain–Barré Syndrome (GBS) and its variant form, Miller Fisher Syndrome (MFS), a chronic and potentially fatal form of paralysis [5, 6].

Unlike most pathogens, *Campylobacter* species show fastidious growth requirements and an unusual sensitivity to environmental stress. *Campylobacter* is considered as an obligate microaerobic bacterium. According to Kaakoush et al. [7], *Campylobacter* requires low oxygen concentration, between 2–10 %, for optimal growth. Moreover, *Campylobacter* seems to be sensitive to high oxygen tensions therefore, theoretically, it cannot grow under an ambient atmosphere, during food processing for instance.

As a microaerophilic bacterium, the main stress encountered by *C. jejuni* is the oxygen concentration in the ambient atmosphere. Under conditions of oxidative stress, oxygen can be reduced to a variety of toxic intermediate products called reactive oxygen species (ROS) including the superoxide anion radical (O_2_^−^), hydrogen peroxide (H_2_O_2_) and the hydroxyl radical (^−^OH) [8]. Oxidative stress is produced when ROS accumulates in the bacteria to a concentration level that exceeds the defense capacity of the cell. However, despite this fastidious requirement, the increasing incidence of human enteric campylobacteriosis indicates that *Campylobacter* has developed mechanisms to survive oxidative stress throughout the whole food chain.

In our laboratory, the clinical *C. jejuni* Bf strain was isolated from a French patient in 1994. No link was established between the illness and the food ingested, but the patient was working in a wastewater treatment plant, suggesting that this strain may be present in the environment. Some studies have evaluated the ability of this strain to enter a viable but non-culturable (VBNC) state [9–12], its increased resistance to oxygen and its ability to grow under an ambient atmosphere (unpublished data). However, no study has been performed to evaluate the resistance to oxygen of *C. jejuni* Bf.

In order to provide data about the oxidative stress resistance of *C. jejuni*, we proposed to characterize the behavior of the clinical *C. jejuni* Bf strain under an aerobic atmosphere and in response to ROS-promoter agents. We evaluated the atypical ability of *C. jejuni* Bf to multiply in an ambient atmosphere, in contrast to several other strains of *C. jejuni* tested. We also studied the morphological changes of this strain under aerobic conditions.

## Results

### Multilocus sequence typing of *C. jejuni* Bf

Using 16S rRNA PCR (Table 1), this isolate was shown to belong to the *Campylobacter* genus (data not shown). Moreover, the MLST analysis revealed that this isolate is a *C. jejuni* strain, which is a member of the clonal complex ST-403.

**Table 1:** Primers used in this study

Primer	Sequence 5′ → 3′	Product size (bp)
*mreB F*	TTCGTACGGCTGGAGATAAG	234
*mreB R*	GTGAAAGAACAGGCGAAGAG	
*rrs F*	AAGGGCCATGATGACTTGACG	207
*rrs R*	AGCGCAACCCACGTATTTAG	
*341 F*	CCTACGGGAGGCACGAG	439
*758 R*	CTACCAGGGTATCTAATCC	
*MD16S F*	ATCTAATGGCTTAACCATTAAAC	856
*MD16S R*	GGACGGTAACTAGTTTAGTATT	
*aspA F*	CCAACTGCAAGATGCTGTACC	625
*aspA R*	GATGGTATTACCGCAAATGAA	
*glnA F*	GCTCAATTCATGGATGGC	722
*glnA R*	GTTCATATGAGCTTATGGAA	
*gltA F*	GTGGCTATCCTATAGAGTGGC	575
*gltA R*	CAGGTATTGGTGCGCTTTGG	
*glyA F*	AGCCTAATTCAGGTTCTCAA	667
*glyA R*	GCGATGGAACGGATAATCACCT	
*pgm F*	TTGGAACTGATGGAGTTCG	1298
*pgm R*	GAAAAAGAAATGAAAAATGTTGTG	
*tkt F*	TGCACCTTTGGGCTTAGC	702
*tkt R*	GCACCTTTGGGTGAAGAAGT	
*uncA F*	AAAGTACAGTGGCACAAGTGG	611
*uncA R*	GCTAGTGATTTAGATGAGGCA	

### Growth/survival of *C. jejuni* strains in microaerobic (MAC) and aerobic conditions (AC)

Cultivation of *C. jejuni* Bf in aerobic conditions in BHI broth using shaking flasks or closed tubes showed large variations in cell numbers in AC (see Additional files 1, 2). These results demonstrate the low reproducibility of *C. jejuni* Bf cultivation under aerobic conditions for suspended cells. A better reproducibility in AC was obtained with cells growing on solid surfaces. Consequently, the growth rate in MAC and AC of the 10 strains was compared using solid surfaces.

To compare the growth and/or survival of *C. jejuni* Bf on solid surfaces according to the gas composition, ten strains of *C. jejuni* were cultured on Karmali agar in AC or MAC for 48 h at 42 °C (Table 2).

**Table 2:** Strains used in this study

Strain of *C. jejuni*	Origin	Clonal complex	RefSeq	References
Bf	Human	ST-403	ND	This study
subsp. *jejuni* NCTC 11168 = ATCC 700819	Human	ST-21	NC_002163.1	Gundogdu et al. [53] Parkhill [54]
RM1221	Chicken	ST-354	NC_003912.7	Fouts et al. [55]
subsp. *jejuni* 81-176	Human	ST-42	NC_008787.1 NC_008790.1 NC_008770.1	Hofreuter et al. [56]
subsp. *doylei* 269.97, RM 4099	Chicken	ND	NC_009707.1	Fouts et al. [57]
subsp. *jejuni* 81116; NCTC 11828	Human	ST-283	NC_009839.1	Pearson et al. [58]
subsp. *jejuni* 00-2538	Human	ST-21	NC_022351.2	Clark et al. [59]
subsp. *jejuni* 00-2544	Human	ST-21	NC_022353.2 NC_022354.1	Clark et al. [59]
subsp. *jejuni* 00-2426	Human	ST-21	NC_022352.2	Clark et al. [59]
subsp. *jejuni* 00-2425	Human	ST-21	NC_022362.2	Clark et al. [59]

*ND* not determined

**Fig. 1:**
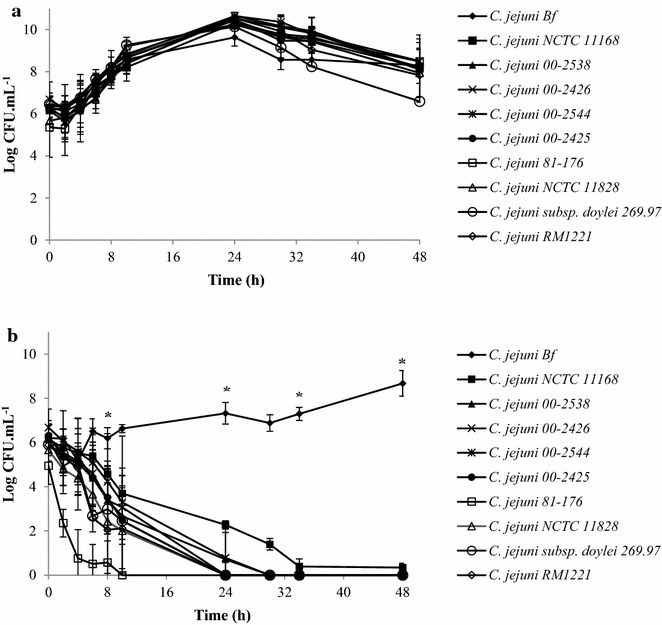
Growth and survival of *C. jejuni* strains under MAC (**a**) and AC (**b**) at 42 °C. The number of colony-forming units was determined by plating serial dilutions on Karmali agar using the microdroplet technique followed by incubation at 42 °C for 24 h in MAC. Plotted points are the mean measurements from three independent experiments. *Error bars* represent standard deviations, although they are too small to be visible in most cases. *Statistically significant difference between the strains in each condition tested (*P* < 0.05)

In MAC, the number of cells increased gradually from approximately 10^6^ CFU mL^−1^ to more than 10^10^ CFU mL^−1^ after 24 h of incubation (Fig. 1a). The same trend was observed for *C. jejuni* Bf strain, with a maximum number of cells of approximately 10^9^ CFU mL^−1^ after 24 h of incubation.

The decrease in the number of cells in MAC was heterogeneous among strains. After 48 h of incubation, the strains showed a decrease in cells to reach approximately 10^8^ CFU mL^−1^, except for *C. jejuni* subsp. *doylei* 269.97, which showed a cell number of 10^7^ CFU mL^−1^ (Fig. 1a).

All strains tested in this study presented a statistically higher specific growth rate under MAC than that of *C. jejuni* Bf strain (Table 3).

**Table 3:** Growth rate after 24 h of incubation (µ_max_) and death times (D-values) in *C. jejuni* strains cultured in MAC and AC

Strain of *C. jejuni*	Rate of growth (µ_max_) in microaerobic conditions	Death times (D-values) in aerobic conditions
Bf	0.5763	\**
subsp. *jejuni* NCTC 11168	0.7714*	0.9737
subsp. *jejuni* 00-2538	0.9198*	1.1039
subsp. *jejuni* 00-2426	0.8964*	1.5442
subsp. *jejuni* 00-2544	0.8303*	1.4554
subsp. *jejuni* 00-2425	0.9085*	1.3785
subsp. *jejuni* 81-176	0.9477*	0.4765
subsp. *jejuni* NCTC 11828	0.7899*	1.3417
subsp. *doylei* 269.97	0.7765*	2.2691
subsp. *jejuni* RM 1221	0.6793*	1.9276

* Statistically significant difference between the strains (*P* < 0.05)

** Not determined because Bf is able to grow under AC

The incubation of the strains under AC led to a decrease in the number of cells of the strains tested, with complete loss of cultivability after approximately 24 h of incubation (Fig. 1b). Only *C. jejuni* NCTC 11168 strain showed a total loss of cultivability after approximately 34 h of incubation under AC. In contrast to the other strains, *C. jejuni* Bf strain in an ambient atmosphere showed a lag phase of around 10 h, which could be identified as an adaptation phase to the stress condition submitted, followed by a 2.5 log cell proliferation (Fig. 1b). Under AC, this strain showed a decrease in cell number after 24 h of incubation and subsequently remained in cell multiplication until the time of analysis (48 h) (Fig. 1b).

**Fig. 2:**
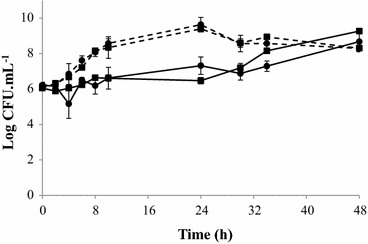
Effect of acclimation to aerobiosis on *C. jejuni* Bf growth under MAC (*dotted line*) and AC (*continuous line*) after aerobiosis acclimation (*square*) or without acclimation (*circle*) at 42 °C. The number of colony-forming units was determined by plating serial dilutions on Karmali agar using the microdroplet technique followed by incubation at 42 °C for 24 h in MAC. Plotted points are the mean measurements from three independent experiments. *Error bars* represent standard deviations, although they are too small to be visible in most cases

Moreover, no significant difference was observed in the number of cells of *C. jejuni* Bf strain cultured in AC and in aerobically acclimated conditions, AAC (Fig. 2). In the two conditions tested, *C. jejuni* Bf showed, on average, an increase of 2.8 log after 48 h of culture in AC at 42 °C (Fig. 2).

The great majority of strains showed death times (D-values) below 1.5 h, with a maximum value of 1.54 h for *C. jejuni* 00-2426 strain and a minimum value of 0.48 h for *C. jejuni* 81-176 strain. However, strains isolated from chicken, *C. jejuni* subsp. *doylei* and RM 1221, showed higher values of death times (2.27 and 1.93, respectively) (Table 3). These results suggest that the strain origin probably influenced the survival capacity under AC, even though only two strains of animal origin were tested.

According to the results obtained in Fig. 1b, and the similar overall response to AC of the different *C. jejuni* strains tested, we selected *C. jejuni* NCTC 11168 for further comparative analyses with *C. jejun*i Bf.

### Cell morphology of *C. jejuni* NCTC 11168 and *C. jejuni* Bf in aerobic conditions

To characterize the effect of oxidative stress on the modification of the cell format of *C. jejuni*, its cell morphology was assessed.

**Fig. 3:**
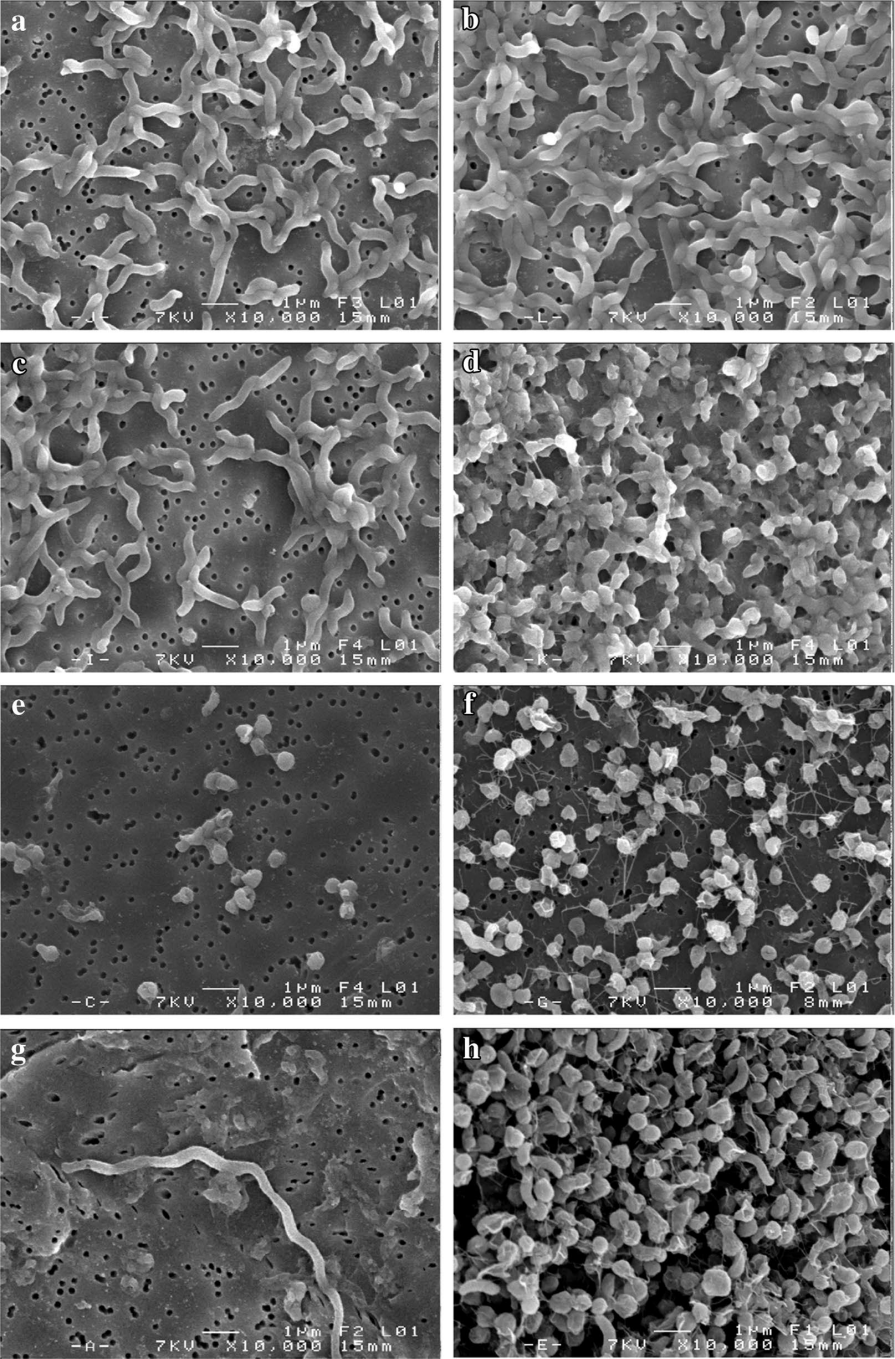
Micrographs of *C. jejuni* NCTC 11168 and *C. jejuni* Bf in MAC (**a**, **b**, respectively), and AC (**c**, **d**, respectively) after 12 h of incubation, and *C. jejuni* NCTC 11168 and *C. jejuni* Bf in MAC (**e**, **f**, respectively), and AC (**g**, **h**, respectively) after 24 h of incubation using SEM on polycarbonate membranes. Magnification: ×10,000

After 12 h of incubation in MAC, the cellular morphology of *C. jejuni* NCTC 11168 and *C. jejuni* Bf exhibited an elongated spiral shape as observed using SEM (Fig. 3) with a size of approximately 2.5 and 1.5 µm, respectively (Fig. 3a, b), while after 12 h of incubation under AC, *C. jejuni* Bf showed a reduced cell size (approximately 0.5 µm), with a predominance of coccoid cells (Fig. 3d). Despite this oxidative stress condition, cells of *C. jejuni* NCTC 11168 kept their spiral shape, with a reduction in cell size to 2.0 µm (Fig. 3c).

An incubation of 24 h under MAC led to a coccoid shape (approximately 0.5 µm) of the *C. jejuni* NCTC 11168 and Bf strains (Fig. 3e, f). *C. jejuni* Bf maintained this shape when it was exposed to an ambient atmosphere, unlike *C. jejuni* NCTC 11168, which exhibited elongated filaments, approximately 20.0 µm in size (Fig. 3g, h). Moreover, under both atmosphere conditions with an incubation of 24 h, cells of *C. jejuni* Bf seemed to interact by close contact and fiber-like structures, like a cobweb (Fig. 3f, h).

### Effect of oxidative stress on the expression of the *mreB* gene in *C. jejuni* NCTC 11168 and *C. jejuni* Bf

The role of oxidative stress in the regulation of the *mreB* gene in *C. jejuni* was assessed at the transcript level, using qRT-PCR.

After 24 h of incubation under MAC, the *mreB* gene expression in the strain *C. jejuni* NCTC 11168 fell by 94.1 % compared with 12 h of incubation while *mreB* expression in *C. jejuni* Bf was reduced by 90.0 %. The expression of *mreB* under AC after 24 h of incubation in *C. jejuni* NCTC 11168 and *C. jejuni* Bf showed an increase in the relative expression of 13.9 and 0.8 times, respectively, when compared with 12 h of incubation.

**Fig. 4:**
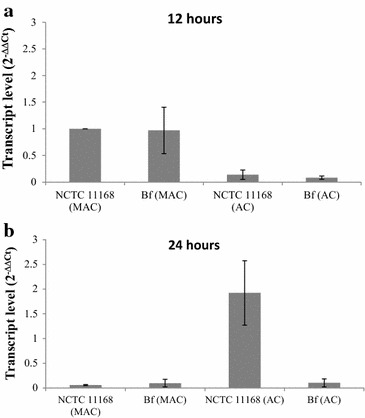
Transcript levels of the *mreB* gene in *C. jejuni* NCTC 11168 and *C. jejuni* Bf in AC and MAC at 12 h (**a**) and 24 h (**b**). Gene expression was estimated using RT-qPCR and the comparative critical threshold (ΔΔC*T*) method. The *rrs* gene was used as the internal control. The level of transcription of the *C. jejuni* NCTC 11168 strain in MAC was used to standardize the analysis

After 12 h of incubation under AC, *mreB* gene expression significantly decreased in both strains tested in comparison with MAC. In fact, *C. jejuni* NCTC 11168 and *C. jejuni* Bf cultured aerobically had a reduced *mreB* gene expression of 86.3 and 91.7 %, respectively (Fig. 4a).

However, after 24 h of incubation, the *C. jejuni* NCTC 11168 strain grown aerobically showed a 1.9-fold increase in the relative *mreB* gene expression when compared to MAC for 12 h. On the other hand, *C. jejuni* NCTC 11168 and *C. jejuni* Bf grown in MAC and *C. jejuni* Bf grown in AC presented a reduced expression of the gene of 94.1, 90.3 and 89.7 %, respectively (Fig. 4b).

### Effects of oxidative stress reagents on the growth of *C. jejuni*

To evaluate the resistance to oxidative stress and the aerotolerance of *C. jejuni*, NCTC 11168 and Bf strains were exposed to oxidative stress reagents (hydrogen peroxide—H_2_O_2_ and paraquat—PQ) in different concentrations.

**Fig. 5:**
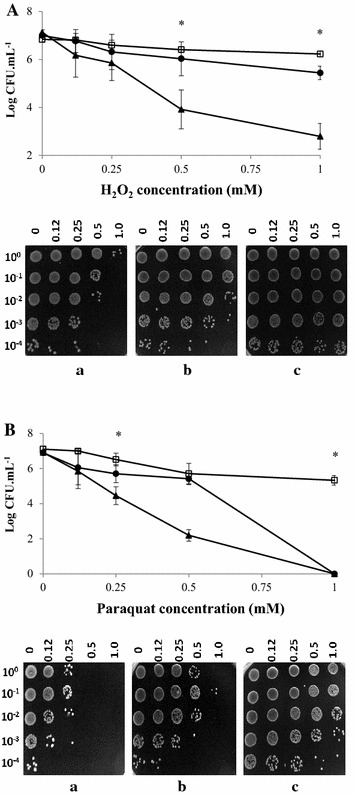
Effect of oxidative stress on the survival of *C. jejuni* NCTC 11168 (*triangle* *a*) and *C. jejuni* Bf (*circle* *b*) strains cultured in MAC, and *C. jejuni* Bf in AAC (*square* *c*) after exposure to different concentrations of H_2_O_2_ (**A**), and paraquat (**B**) for 1 h at 42 °C. The number of colony-forming units was determined by plating serial dilutions on Karmali agar using the microdroplet technique followed by incubation at 42 °C for 24 h in MAC. The viability of oxidative stress was determined by inoculating 10 µL of bacterial suspension on Karmali agar plates after exposure to different concentrations of H_2_O_2_ and paraquat followed by incubation at 42 °C for 24 h in MAC. Plotted points are the mean measurements from three independent experiments. *Error bars* represent standard deviations, although they are too small to be visible in most cases. *Statistically significant difference between the strains in each condition tested (*P* < 0.05)

*Campylobacter jejuni* Bf strain cultivated in MAC or AAC showed a higher survival rate in the presence of H_2_O_2_ and PQ than *C. jejuni* NCTC 11168 strain (Fig. 5).

Indeed, *C. jejuni* Bf strain cultivated in MAC and AAC exhibited a higher survival rate of 38.6 and 52 %, respectively, in the presence of H_2_O_2_ (1 mM) compared to the reference strain *C. jejuni* NCTC 11168 (Fig. 5A). The two *C. jejuni* strains evaluated showed no significant difference in survival at concentrations below 0.5 mM H_2_O_2_.

In the presence of 0.5 mM paraquat, *C. jejuni* NCTC 11168 strain showed a decrease of 4.7 log, while the *C. jejuni* Bf strain cultivated in MAC or AAC showed a limited reduction of 1.5 and 1.4 log, respectively. Concentrations above 1 mM PQ were lethal for the reference strain but not for aerobically-acclimated *C. jejuni* Bf (Fig. 5B).

## Discussion

In this study, some phenotypic and genotypic characteristics of the atypical *C. jejuni* Bf strain were investigated, in order to provide data about the aerotolerance of this strain. It was shown that this strain can grow under aerobic conditions (AC), unlike other strains of *C. jejuni*. Oxidative stress changes the morphology of this strain and the transcription of the *mreB* gene, responsible for maintaining the bacillary cell morphology, is down-expressed. Moreover, *C. jejuni* Bf strain is less susceptible to oxidative stress mediated by ROS-promoter agents, in comparison with the other *C. jejuni* strains tested. Taken together, these data highlight the resistance to oxidative stress conditions of *C. jejuni* Bf.

The clonal complex ST-403, to which the *C. jejuni* Bf strain belongs, has been isolated from humans [13–15], but the origins of this complex are unclear, although it has been found in porcine isolates and occasionally in cattle [16–18]. This clonal complex is associated with Guillain–Barré Syndrome or enteritis as indicated by ST-403 *C. jejuni* HS:23 isolated from patients with these diseases [19].

As a microaerophilic microorganism, *C. jejuni* requires 2–10 % O_2_ for optimal growth [7]. However, we report here that the *C. jejuni* Bf strain can survive and multiply under certain AC, unlike several other strains of *C. jejuni*. Although some studies have already reported the ability of *C. jejuni* strains to survive oxygen [7, 19, 20], this is the first report demonstrating the capacity of a *C. jejuni* strain to multiply under atmospheric conditions, with a significant and continuous growth. Further studies are required to define whether this aero-tolerant feature observed in the current study is specific to *C. jejuni* Bf or could be generalized. Furthermore, this property could explain the persistence and spread of this organism in the food chain.

Many studies have investigated the survival of *C. jejuni* under oxidative stress. Chynoweth et al. [20] observed that strains of *C. jejuni* isolated from different sources showed the ability to adapt in atmospheric oxygen. Growth was limited to a few colonies on nutrient agar in atmospheric oxygen. Kaakoush et al. [7] evaluated the growth of *C. jejuni* strain NCTC 11168 at several cell densities in BHI broth cultures. As no growth under AC using initial cell densities lower than 10^4^ CFU mL^−1^ and growth in a 19 % O_2_ atmosphere with *Campylobacter* densities higher than 10^5^ CFU mL^−1^ were observed, they concluded that oxygen plays a growth-limiting role in high-density bacterial cultures under MAC and is toxic in low-density cultures under AC in C. *jejuni* NCTC 11168 [7]. However, the experiments conducted by Kaakoush et al. [7] were performed in liquid medium. Notably, oxygen transfer in liquid medium is low (*k*_H_ = 9.28 × 10^−4^ mol L^−1^ atm^−1^ in water at 42 °C) and the oxygen demand by the microorganism with cell densities around 10^5^–10^6^ CFU mL^−1^ is higher than for lower densities (lower than 10^4^ CFU mL^−1^). These two factors lead to a decrease in the dissolved oxygen concentration in liquid medium, which can induce the growth of *Campylobacter* in AC. In addition, we observed a low reproducibility of *C. jejuni* Bf cultivation under AC in liquid medium (BHI). Due to the absence of a robust biological reproducibility of these results, the growth curve in AC and MAC was performed on a solid surface using Karmali agar. Growth on the agar plate was reproducible for all strains used in the conditions tested, demonstrating the validity of the method. Although not a routine technique, the microbial surface growth of different strains has previously been carried out by many researchers [21–25]. Furthermore, Fujikawa and Morozumi [26] observed that the growth curves of *Escherichia coli* on a nutrient agar plate at constant temperatures were very similar to those in a liquid.

Jones et al. [19] reported that the *C. jejuni* 13997 strain grown under MAC on blood agar plates and subsequently stored under AC survived better than on plates stored under MAC. These results suggest the ability of this *C. jejuni* strain to adapt to an ambient gaseous atmosphere. However, these authors observed only a better survival of the *C. jejuni* strain tested, but no cellular growth under AC [19].

The viability of the reference strains of *C. jejuni* (NCTC 11168, NCTC 11828 and 81-176) under an atmospheric level of oxygen has also been evaluated in the literature [27–29]. After 9 h of exposure under atmospheric conditions, the viability of *C. jejuni* NCTC 11828 and *C. jejuni* 81-176 decreased to 10 and 50 %, respectively [28, 29] while *C. jejuni* NCTC 11168 exposed to ambient oxygen at 37 °C showed a reduction of approximately 2 log after 48 h of culture [27]. In the present study, *C. jejuni* NCTC 11828 and 81-176 strains presented a reduction of 54.4 and 88.6 % viability after 8 h of exposure to atmospheric oxygen, respectively, and *C. jejuni* NCTC 11168 showed a greater reduction (5.8 log) after 48 h of culture in AC at 42 °C.

In order to verify if the ability to grow in certain AC is due to an increase in oxidative stress resistance, we tested the behavior of *C. jejuni* Bf in the presence of H_2_O_2_ and paraquat. *C. jejuni* Bf was less susceptible to stress conditions induced by H_2_O_2_ and paraquat than the reference strain *C. jejuni* NCTC 11168, for which the lethal effect of paraquat was concentration-dependent. Unlike the results observed with *C. jejuni* Bf, previous studies have shown the sensitivity of *C. jejuni* referent strains to oxidative stress induced by H_2_O_2_ and paraquat. Garénaux et al. [30] observed that 1 h of exposure to 500 μM paraquat led to a 0.5 log_10_ reduction in the *C. jejuni* NCTC 11168 population. This same strain exposed to the final concentration of 1 mM H_2_O_2_ and paraquat for 1 h showed reduced viability (0.5 and 0.1 %, respectively) at 42 °C under MAC [31]. This resistance to oxidative stress induced by ROS-promoter agents has also been observed in other strains of *C. jejuni* [13, 29].

In the presence of oxygen, the microorganism undergoes genetic and physiological changes in response to oxidative stress. Some studies have reported a transition from the spiral to the coccoid form in cells of *C. jejuni* submitted to oxidative stress conditions [32, 33]. In addition, *C. jejuni* cells may be seen as filaments, doughnuts and straight rods [34–36]. According to Griffiths [35], the rod form of *C. jejuni* is considered to be the usual viable form in the exponential growth phase, whilst filaments and coccoid forms are associated with the stationary growth phase. We have confirmed this phenotype in *C. jejuni* Bf and *C. jejuni* NCTC 11168 submitted to oxidative stress conditions. According to our results, the stationary growth phase (after 24 h of incubation) and particularly oxidative stress conditions are the stimuli for cell transition from the spiral to the coccoid form. The presence of fiber-like structures in *C. jejuni* Bf cells under AC and MAC after 24 h of incubation probably plays a role in protecting them against oxidative stress [37]. Other studies have obtained similar results of cell morphology modification from spiral to coccoid forms after exposure to atmospheric oxygen [38, 39].

Cell size reduction and transition to coccoid forms might be associated with a modification of *mreB* gene expression, responsible for maintaining the bacillary cell morphology by forming helical filaments underneath the cell membrane [40–44]. It was suggested that the *mreB*-like gene present in the genome of *C. jejuni* NCTC 11168 plays a similar role in the maintenance of the bacterial cell shape [33]. The reduction in *mreB* expression in *C. jejuni* Bf and NCTC 11168 shown in this work during oxidative stress and stationary growth phase most likely leads to the morphological change in the cells under stress conditions. In *E. coli*, *Bacillus subtilis* and *Caulobacter crescentus*, the inactivation of *mreB* genes resulted in coccoid form formation [42, 45, 46]. Transcriptional down-regulation of *Helicobacter hepaticus mreB* and *mreC* genes also induced the formation of spherical cells [47].

This study clearly demonstrates that exposure to oxidative stress plays an important role in the morphological changes of *C. jejuni* under AC. These changes could contribute to the adaptation and survival of these strains in stressful conditions. Although the Bf strain exposed to AC presents a large amount of coccoid cells, this strain is able to multiply. This could be due to the presence of shorter spiral forms, which have previously been observed after exposure to atmosphere oxygen concentration [38].

## Conclusions

In conclusion, *C. jejuni* Bf can grow and multiply under AC, unlike other strains of *C. jejuni* and showed a great resistance to compounds that induce oxidative stress, such as hydrogen peroxide and paraquat. This strain is therefore more adapted to survive oxidative stress conditions. During the transmission of *C. jejuni* to food preparation surfaces, the resistance of this strain to oxidative stress may be a key factor in explaining its spread and survival in the environment. The more this pathogen can survive in its environment, the greater is the probability of being exposed to this hazard.

However, it is important to note that we cannot say whether this property of oxidative stress resistance is intrinsic to or recently gained by a sub-type of *C. jejuni*, considering that only a total of ten strains of *C. jejuni* were tested. In addition, further studies are required to determine the cellular mechanisms that contribute to the resistance of this strain to oxidative stress. Moreover, the impact on different biological processes of *C. jejuni* Bf growth capacity under AC, such as its persistence in the environment, its virulence, or biofilm formation, could be investigated.

## Methods

### Bacterial strains and growth conditions

Strains used in this study are presented in Table 2. The genomes of the selected strains are entirely sequenced and available on NCBI. The cultures were stored at −80 °C in microtubes containing Brain–Heart Infusion medium (BHI) containing 20 % sterile glycerol. Before each experiment, the strains were grown at 42 °C on Karmali agar plates (Oxoid, Dardilly, France) for 48 h under microaerobic conditions, namely MAC (10 % CO_2_, 5 % O_2_ and 85 % N_2_) obtained using gas replacement jars (GRJ) 4× flushed/filled with a MACS MICS system (Whitley jar gassing system) at −50 kPa vacuum.

To prepare cells cultured in aerobic conditions (ambient atmosphere), namely AC, the strains were grown first in MAC at 42 °C on Karmali agar for 48 h. They were then harvested and resuspended in peptone saline solution and standardized to an optical density at 600 nm (OD_600nm_) of 0.03 (≈10^6^ CFU mL^−1^). Each bacterial suspension, containing approximately 10^6^ CFU mL^−1^, was then inoculated onto Karmali agar plates and incubated at 42 °C under AC for 24 h.

To prepare cells in aerobically acclimated conditions, namely AAC, *C. jejuni* Bf was first grown in MAC on Karmali agar for 48 h. Then, colonies were recovered and subcultured three times successively on Karmali agar plates followed by incubation at 42 °C for 24 h in AC.

### DNA extraction, 16S rDNA and MLST typing of *C. jejuni* Bf

DNA of *C. jejuni* Bf was extracted using the Wizard^®^ Genomic DNA Purification Kit (Promega, Charbonnières, France) according to the manufacturer’s instructions. DNA integrity and quantity were determined using an Experion™ Automated Electrophoresis System (BioRad, Marnes la Coquette, France) and NanoDrop 2000c (Fisher Scientific, Illkirch, France), respectively. A DNA fragment of 16S rDNA was amplified using primers specific to *C. jejuni* MD16S1 and MD16S2 [48] and sequenced using the Sanger protocol by Biofidal (Vaux-en-Velin, France). MLST was achieved according to Dingle et al. [49]. Briefly, the seven housekeeping genes for MLST (*aspA*, *glnA*, *gltA*, *glyA*, *pgm*, *tkt* and *uncA*) were amplified by PCR using the primers mentioned in Table 1. The PCR products were sequenced by Biofidal (Vaux-en-Velin, France). All allelic sequences were queried against the *C. jejuni* MLST database (http://pubmlst.org/campylobacter) to retrieve sequence types (STs) and determine the clonal complex of *C. jejuni* Bf. The nucleotide sequence data were submitted to the *Campylobacter* PubMLST database for allele assignments.

### Growth curve of *C. jejuni* strains

Growth curves of *C. jejuni* NCTC 11168 (reference strain) and Bf were obtained from liquid cultures (using BHI) and the growth curve of *C. jejuni* strains shown in Table 2 were obtained from solid cultures. Bacteria were cultivated in MAC at 42 °C for 48 h. Then, colonies were transferred into a flask containing 10 mL of BHI broth grown in MAC at 42 °C with shaking (80 rpm) for 24 h. After incubation, aliquots corresponding to 2 % of the cultures (≈10^6^ CFU mL^−1^) of *C. jejuni* NCTC 11168 and *C. jejuni* Bf were transferred to a flask containing 100 mL of BHI broth and incubated at 37 or 42 °C under shaking for 9 h in MAC and AC.

Alternatively, *C. jejuni* NCTC 11168 and Bf were cultivated in MAC at 42 °C for 48 h. Then, colonies were transferred to 50 mL of BHI in a hermetic closed tube in order to prevent a large exchange of oxygen between the culture medium and the environment. The tube was incubated under AC at 42 °C with shaking (80 rpm) for 24 h. After incubation, aliquots containing 10^6^ cells mL^−1^ were inoculated in a flask containing 25 mL of BHI broth and incubated at 42 °C with shaking (80 rpm) for 18 days under AC.

*Campylobacter jejuni* NCTC 11168 and Bf were enumerated on Karmali agar plates using the microdroplet technique [50]. Briefly, 20 µL of serial dilutions were deposited on Karmali agar plates and incubated at 42 °C for 48 h. *C. jejuni* plate counts were expressed in CFU mL^−1^.

To determine the growth curve of *C. jejuni* strains in solid cultures, bacteria were cultivated in MAC at 42 °C for 48 h. However, *C. jejuni* Bf was cultured in AC and AAC as well. The cells obtained in these different conditions were resuspended in peptone saline solution and standardized to an OD_600nm_ of 0.03. Then, cells were plated on Karmali agar plates and incubated at 42 °C in AC and MAC for 48 h. After incubation, cells were recovered in peptone saline solution. *C. jejuni* was enumerated on Karmali agar plates using the microdroplet technique followed by incubation at 42 °C for 48 h in MAC. The experiment was performed in triplicate using three biological replicates. The D-value was calculated according to the formula:

$$\log \left( {\frac{N}{{N_{0} }}} \right) = - \frac{t}{D};$$

where N_0_ is the initial number of cells, N is the number of cells after time t of exposure to oxygen at 42 °C and D is the rate constant for inactivation of cells. Statistically significant differences were calculated using the Student *t* test. Values <0.05 were considered statistically significant.

### Scanning electron microscopy

*Campylobacter jejuni* NCTC 11168 and *C. jejuni* Bf cells were cultivated in MAC at 42 °C for 48 h. Then, cells were resuspended in peptone saline solution, and standardized to an OD_600nm_ of 0.01 (≈10^4^ CFU mL^−1^). The cells were plated onto Karmali agar and incubated at 42 °C in AC and MAC for 12 and 24 h. After incubation, cells were recovered in peptone saline solution and aliquots of the cell suspensions were filtered on a white polycarbonate membrane (Millipore) with a 0.22 µm pore size. The membrane containing the specimens was fixed with 2.5 % (v/v) glutaraldehyde solution for 48 h at 4 °C. Then, the samples were washed in 0.2 mol L^−1^ sodium cacodylate (pH 7.2), and dehydrated in serial concentrations of ethanol. The membranes were transferred to a critical point dryer, and the specimens were subsequently sputter-coated and observed under a scanning electron microscope (Jeol JSM 6301F).

### Bacterial RNA isolation and purification

Expression of the *mreB* gene, responsible for maintaining the bacillary cell morphology of the bacteria [40, 41, 51], was compared between MAC and AC. Bacterial RNA was obtained from *C. jejuni* NCTC 11168 and *C. jejuni* Bf strains cultured for 12 and 24 h in AC and MAC, at 42 °C.

After the incubation time, RNA Protect Reagent (Qiagen, Hilden) was added to the cells. Cells were centrifuged at 3300×*g* for 6 min at 4 °C, and then resuspended in 1 mL of Extract-All (Eurobio, France) and 0.2 mL of chloroform. After centrifugation at 12,000×*g* for 15 min at 4 °C, the aqueous phase was removed. Total RNA was precipitated in isopropanol, rinsed in cold ethanol 75 %, then solubilized in 50 μL of Rnase-free water (Qiagen, Hilden). DNA was removed by digestion using RQ1 RNase-free DNase (Promega, USA), and then total DNA removal was verified by PCR using 341F/758R primers. The quality and quantity of RNA were checked using a NanoDrop spectrophotometer (NanoDrop^®^ 2000, Thermo Scientific). The integrity of RNA samples was verified on a 1 % agarose gel; electrophoresis was carried out in Tris–acetate–EDTA buffer for 50 min at 90 V. RNA concentrations were then standardized to 100 ng for each sample prior to reverse transcription.

### Quantitative real-time reverse transcription PCR

The cDNA synthesis was performed using the RevertAid H Minus First-Strand cDNA synthesis kit (Euromedex) with the random hexamer primer according to the manufacturer’s instructions. The quantitative real-time PCR assay was performed with SYBR Green I (Applied Biosystems, USA) using an MJ Research PTC-200 Thermal Cycler and *mreB* and *rrs* primers (Table 1). The *rrs* gene was used as the internal control [52]. The composition of the PCR mix was as follows: 5.0 μL of sample, reverse primer (1 μM), forward primer (1 μM), and 12.5 μL of SYBR Green I Master Mix. The amplification program included an initial denaturing step at 95 °C (10 min), followed by 40 cycles at 95 °C (15 s) and 60 °C (1 min). A negative control (without cDNA) was included in each run. Relative quantification of *mreB* expression was calculated according to the 2^−ΔΔCt^ method. The experiments were performed in triplicate from three independent cultures.

### Survival of *C. jejuni* NCTC 11168 and *C. jejuni* Bf in oxidative stress conditions

*Campylobacter jejuni* NCTC 11168 and *C. jejuni* Bf grown in MAC and *C. jejuni* Bf in AAC were resuspended in peptone saline solution, and standardized to an OD_600nm_ of 0.03. Subsequently, hydrogen peroxide (H_2_O_2_) (Sigma-Aldrich, Saint-Quentin Fallavier, France) or paraquat (PQ) (MP Biomedicals, Illkirch, France) solutions were used to induce a superoxidative and a peroxide stress, respectively. Concentrations of 0.12, 0.25, 0.50 and 1 mM of H_2_O_2_ or PQ were added to the bacterial suspension, which were incubated at 42 °C for 1 h under MAC. The ability of the strains to resist oxidative stress was assessed by plating serial dilutions on Karmali agar plates using the microdroplet technique followed by incubation at 42 °C for 48 h under MAC. The experiment was performed in triplicate from three independent cultures. Statistically significant differences were calculated using the Student *t* test. *P* values <0.05 were considered statistically significant.

## Additional files

10.1186/s13099-015-0077-x Growth and survival of *C. jejuni* NCTC 11168 (●) and Bf (■) under MAC (continuous lines) and AC (dashed lines) at 37°C (A) and 42°C (B) in BHI broth. The number of colony-forming units was determined by plating serial dilutions on Karmali agar using the microdroplet technique followed by incubation at 42°C for 24 h in MAC. Plotted points are the mean measurements from three independent experiments. Error bars represent standard deviations, although they are too small to be visible in some cases.
10.1186/s13099-015-0077-x Growth and survival of *C. jejuni* NCTC and Bf in AC at 42°C in BHI broth.
