# Supplementary material for: Description of Campylobacter jejuni Bf, an atypical aero-tolerant strain
Source: Gut Pathog. 2015 Nov 19;7:30. doi: 10.1186/s13099-015-0077-x (PMC4653858; doi:10.1186/s13099-015-0077-x)
Supplement: Supplementary file 1 — 10.1186/s13099-015-0077-x Growth and survival of C. jejuni NCTC 11168 (●) and Bf (■) under MAC (continuous lines) and AC (dashed lines) at 37°C (A) and 42°C (B) in BHI broth. The number of colony-forming units was determined by plating serial dilutions on Karmali agar using the microdroplet technique followed by incubation at 42°C for 24 h in MAC. Plotted points are the mean measurements from three independent experiments. Error bars represent standard deviations, although they are too small to be visible in some cases. [file 13099_2015_77_MOESM1_ESM.docx]

| **A)** |
| --- |
| **B)** |
