# Supplementary material for: Description of Campylobacter jejuni Bf, an atypical aero-tolerant strain
Source: Gut Pathog. 2015 Nov 19;7:30. doi: 10.1186/s13099-015-0077-x (PMC4653858; doi:10.1186/s13099-015-0077-x)
Supplement: Supplementary file 2 — 10.1186/s13099-015-0077-x Growth and survival of C. jejuni NCTC and Bf in AC at 42°C in BHI broth. [file 13099_2015_77_MOESM2_ESM.docx]

**Additional file 2.** Growth and survival of *C. jejuni* NCTC and Bf in AC at 42°C in BHI broth.

| Time (days) | *C. jejuni* NCTC 11168 | | *C. jejuni* Bf | |
| --- | --- | --- | --- | --- |
|  | Log CFU. mL^-1^  Media | Standard deviation (SD) | Log CFU. mL^-1^  Media | Standard deviation (SD) |
| 0 | 6.3 | 0.1414 | 6.5 | 0,1 |
| 3 | <0.1 | - | 5.6 | 1.9157 |
| 4 | <0.1 | - | 5.7 | 1.9655 |
| 5 | <0.1 | - | 4.7 | 4.1103 |
| 6 | <0.1 | - | 4.3 | 4.3000 |
| 8 | <0.1 | - | 2.0 | 2.2913 |
| 9 | <0.1 | - | 1.4 | 2.4249 |
| 12 | <0.1 | - | 1.9 | 3.2332 |
| 13 | <0.1 | - | 1.8 | 3.1177 |
| 18 | <0.1 | - | <0.1 | - |
